# Jürgen W. Spranger, Paula W. Brill, Christine Hall, Gen Nishimura, Andrea Superti-Furga, and Sheila Unger: *Bone dysplasias: an atlas of genetic disorders of skeletal development*: Fourth Edition, ISBN: 9780190626655

**DOI:** 10.1007/s00439-019-02038-0

**Published:** 2019-06-10

**Authors:** Sarah Smithson

**Affiliations:** Bristol, UK

The fourth edition of Bone Dysplasias provides an excellent reference for professionals working in the field of skeletal dysplasia, including geneticists, radiologists, paediatricians, orthopaedic surgeons and therapists. There is a short introduction focusing on the history of skeletal dysplasias which is helpful to establish the context of the current atlas and understand the long journey leading to its publication.

The format of the book is simple to follow, so that the reader can easily locate the specific information they want. Each condition has a short dedicated section with a summary of the major clinical and radiological findings. Then, there follows a section on genetics, with mode of inheritance and the underlying genetic basis. There are also sections on clinical course and prognosis as well as some remarks which are particularly helpful, as they condense the experience and wisdom of the authors, who have worked in the field for many years. The written content is very succinct, so that a great deal of information may be extracted by the reader in a short time. Each section is illustrated with X-ray images, including different anatomical regions at various ages from birth to adult life. This very important feature allows appreciation of the evolving characteristics of the condition over time and is a useful source for comparison with patient X-rays when trying to establish a diagnosis in the clinic. For some conditions, there are clinical photographs which allow for correlation between physical appearance or dysmorphic features and radiological findings. The legends are very useful in describing in detail the particular radiological finding at each stage of skeletal development. The atlas approach is accessible for those who are experienced in the field and for new learners with little prior experience of skeletal disorders. Key references for each condition are also provided, which point the reader to further and more detailed texts.

A major change since the previous edition is in the way the skeletal dysplasias are classified in chapters in the book. In the third edition, the 13 chapters grouped the dysplasias according to severity, such as lethal conditions, or a particular characteristic, such as increased bone density. In the new edition, there are 26 chapters which follow the structure of the published Nosology of Skeletal Dysplasias, in which they are grouped according to the gene, protein, or cellular pathway as much as possible. This provides consistency for the reader and is likely to enhance learning as they return to the chapters repeatedly over time. Furthermore, this approach emphasises the relationship between conditions that are of variable severity, but are caused by the same gene and the reader can recognise the spectra of clinical and radiological findings. For example, in Chapter 4, the reader who may not have appreciated the link between metatropic dysplasia and brachyolmia through their different clinical presentations will observe the overlap between *TRPV4*-related conditions which are so well illustrated.

For colleagues who are new to the Skeletal Dysplasia field and for experts, this book is highly recommended. It will provide an invaluable source of information and will be a useful companion in the clinic.

